# Factors for Predicting Instant Neurological Recovery of Patients with Motor Complete Traumatic Spinal Cord Injury

**DOI:** 10.3390/jcm11144086

**Published:** 2022-07-14

**Authors:** Xiangcheng Gao, Yining Gong, Bo Zhang, Dingjun Hao, Baorong He, Liang Yan

**Affiliations:** 1 Department of Spine Surgery, Honghui Hospital, Xi’an Jiaotong University, Xi’an 710054, China; gxc071@yau.edu.cn (X.G.); gong_yn@bjmu.edu.cn (Y.G.); 1710301250@bjmu.edu.cn (B.Z.); msk@bjmu.edu.cn (D.H.); 1510301104@pku.edu.cn (B.H.); 2 Medical College, Yan’an University, Yan’an 716000, China

**Keywords:** traumatic spinal cord injury, neurological recovery, factors, in-patients

## Abstract

The objective of this study was to analyze the factors affecting the instant recovery of neurological function in patients with motor complete traumatic spinal cord injury (TSCI) treated in hospital. **Methods:** A retrospective analysis of 1053 patients with TSCI classified according to the American Spinal Cord Injury Association (ASIA) as grades A and B at 59 tertiary hospitals from 1 January 2018 to 31 December 2018 was performed. All patients were classified into motor complete injury (ASIA A or B) and motor incomplete injury (ASIA C or D) groups, according to the ASIA upon discharge. The injury level, fracture segment, fracture type, ASIA score at admission and discharge, treatment protocol, and complications were recorded. Univariate and multivariate analyses were performed to evaluate the relationship between various factors and the recovery of neurological function. **Results:** The results of multiple logistic regression analysis revealed that the ASIA score on admission (*p* < 0.001, odds ratio (OR) = 5.722, 95% confidence interval (CI): 4.147–7.895), fracture or dislocation (*p* = 0.001, OR = 0.523, 95% CI: 0.357–0.767), treatment protocol (*p* < 0.001; OR = 2.664, 95% CI: 1.689–4.203), and inpatient rehabilitation (*p* < 0.001, OR = 2.089, 95% CI: 1.501–2.909) were independently associated with the recovery of neurological function. **Conclusion:** The recovery of neurological function is dependent on the ASIA score on admission, fracture or dislocation, treatment protocol, and inpatient rehabilitation.

## 1. Introduction

Traumatic spinal cord injury (TSCI), one of the most severe traumatology injuries, is a common occurrence globally, resulting in severe, including permanent, disability due to motor, sensory, and autonomic dysfunction [1,2]. The incidence of TSCI varies among countries, ranging from 3.6 to 195.4 patients per million around the world [3,4]. The consequences for the physical, social, and vocational well-being of patients are devastating, due to the consequent loss of independence, high incidence of complications, and considerable medical cost [5]. The treatment of TSCI includes both surgical and nonoperative treatments. Timely diagnosis and protection of nerves should be the primary considerations in the treatment of TSCI in order to save the gradual loss of functional nerve tissue. Key neuroprotective interventions include decompressive surgery, methylprednisolone administration, and hemodynamic changes [6].

Although great progress has been made in the operative and nonoperative treatment of TSCI in the past three decades, the recovery of neurological function is still one of the most concerning issues for patients and doctors [7,8]. Patients after TSCI usually ask some questions regarding function: “Will I walk again?” and “What will I be able to do?” Furthermore, in countries with insurance-based healthcare systems, the medical officers have to justify and fight for appropriate services; furthermore they have to know how to allocate resources. Therefore, predicting the potential for neurological recovery in patients with TSCI, particularly in those with motor complete injury, is important for guiding treatment, setting realistic goals, planning optimized rehabilitation, and addressing questions from patients and their relatives. To date, the recovery of neurological function has been discussed in several studies [9,10,11]. However, most studies have focused on factors influencing long-term neurological function recovery [12,13]. Few studies have focused on the factors affecting early neurological function recovery.

Therefore, it is necessary to predict functional outcome after TSCI, especially during the early stage after motor complete traumatic spinal cord injury in order to plan treatment and rehabilitation in subsequent stages [14], because the instant functional recovery not only indicates the long-term functional outcome, but also affects patients’ trust in doctors, doctor–patient relationship, confidence recovery, and subsequent treatment compliance [8]. In addition, Kirshblum et al. reported a strong correlation between early and long-term neurological function recovery, and concluded that examinations completed between 72 h and 1 week after injury are optimal for research and prognostication [15]. Unfortunately, previously published studies were mostly based on retrospective studies with small samples, and the medical environment at home and abroad may affect the results [9,14,16]. Therefore, we aimed to analyze the relative factors regarding instant neurological recovery of patients with motor complete injury using data from multiple centers within a short period.

## 2. Materials and Methods

### 2.1. Ethical Approval

The study was approved by the institutional review board of Honghui Hospital, affiliated with Xi’an Jiaotong University (No. 201904001), and the need for informed consent was waived due to the retrospective nature of the study and the absence of any intervention. Furthermore, we only collected patients’ medical data for clinical analysis without obtaining videos or photos. The medical data collected will not be used for nonresearch purposes, and all identifying information will be deleted for publication.

### 2.2. Study Population

We retrospectively collected data from the medical records and radiological materials of patients with TSCI at 59 spine centers in China, from 1 January 2018 to 31 December 2018, and participants were selected using the International Classification of Diseases Version ICD-10-CM and the diagnostic code for TSCI (Supplementary Material S1). ASIA was used to classify the completeness of the spinal cord injury. In brief, motor complete injury (ASIA grade A) is defined as loss of sensory and motor function below the level of injury, including at the S4–S5 level. Sensory incomplete injury (ASIA grade B) is defined as loss of motor function, including an inability to contract the anal sphincter, with some spared sensation below the level of injury.

The inclusion criteria were as follows: (1) confirmed diagnosis of TSCI, (2) motor complete injury (ASIA A or B) upon admission, (3) new-onset injury within 7 days, (4) neurological level from C1 to L1 affected, and (5) hospitalization. Exclusion criteria were as follows: (1) nontraumatic spinal cord injury, (2) motor incomplete injury (ASIA C or D) or intact neurological status (ASIA E), (3) death before discharge, (4) attending emergency department without hospitalization, and (5) incomplete data. All enrolled patients were classified into motor complete injury (ASIA A or B) or motor incomplete injury (ASIA C or D) groups when discharged.

### 2.3. Data Recording Form

We designed an inpatient data recording form, referring to relevant global epidemiological research on TSCI, epidemiological experts, and clinical experts at the Xi’an Honghui Hospital (Supplementary Material S2). According to the rationality, scientific nature, necessity, and feasibility of the investigation scheme, several rounds of argumentation, modification, and field tests were conducted until reliable information was collected. We then created a final version of the data recording form and created a database. The established database included the following indicators: (1) general information and demographic characteristics (name, age, sex, ID number, and occupation); (2) relevant information about injury (date of injury, cause of injury, date of admission, injury level, severity of injury, and spinal fracture); (3) ASIA scores on admission and at discharge; (4) treatment protocol (surgery or non-surgery, surgical approach, and procedures); (5) complications during the hospital stay; (6) rehabilitation; and (7) in-hospital death.

### 2.4. Statistical Analysis

Epidata version 3.1 (Epidata Association, Odense, Denmark) was used to enter the data, and Microsoft Office Excel 2019 (Microsoft Corporation, Redding, CA, USA) was used to check and save the data. All analyses were conducted using SPSS version 26 (IBM, Armonk, NY, USA) and GraphPad Prism version 9.3.0 (San Diego, CA, USA). Continuous data are expressed as medians and ranges, and nominal data are expressed as frequencies and percentages. Logistic regression models were fitted to calculate the odds ratios (ORs) and 95% confidence intervals (CIs) of the related factors for instant neurological recovery. Univariate logistic regression analyses were performed to examine the associations of age, sex, mechanism of trauma, injury level, in-hospital complications, fractures or dislocations, treatment protocol, surgical procedures, American Spinal Injury Association (ASIA) score on admission, inpatient rehabilitation, and the time before admission. Multivariate logistic regression analysis was performed to determine the factors associated with instant neurological recovery. Variables that exhibited a significant difference in the univariate analysis were analyzed using multivariate logistic regression. The predefined significance level for inclusion in the regression model was a *p*-value of 0.05. The results are reported as odds ratios (ORs) with 95% confidence intervals (CIs) and *p*-values.

## 3. Results

### 3.1. General Characteristics of Patients

The study involved 59 tertiary hospitals in China and a total of 1053 patients (men: 851 (80.8%), women: 202 (19.2%), median age: 50 (range: 2–92) years old) were included. The proportions of patients aged <50 and ≥50 years were 56.5% (595/1053) and 43.5% (458/1053), respectively. The median time before admission was 24 h (ranged from 0 to 168 h). There were 748 (71.0%) and 305 (29.0%) patients whose time before admission was <24 h and ≥24 h, respectively. The duration of hospital stay until ASIA assessment upon discharge ranged from 0 to 180 days (mean (standard deviation): 22.5 (17.8) days).

Regarding the level of TSCI, the most common was cervical spinal cord injury (571 cases), which accounted for 54.2% of all cases, followed by thoracic and lumbar injuries (254 and 228 cases, respectively). Falls from heights (416/1053, 39.5%), vehicle accidents (257/1053, 24.4%), and tumbles (207/1053, 19.6%) were the three most common mechanisms of trauma. Regarding the injury level, 571 (54.2%), 254 (24.1%), and 228 (21.7%) patients had cervical spinal cord, thoracic spinal cord, and lumbosacral spinal cord injuries, respectively. ASIA scores on admission were recorded, and ASIA A (70.3%, 740 cases) accounted for the highest proportion of patients with TSCI, followed by ASIA B (29.7%, 313 cases). A total of 79.8% (840 cases) of the TSCI patients had spinal fractures or dislocations. A total of 47.8% (503 cases) of the patients with TSCI experienced clinical complications; 822 (78.1%) patients underwent surgical treatment; and the main surgical procedures were decompression, fixation, and bone graft fusion in 543 (51.6%) patients. Three hundred and sixty-two patients (36.2%) underwent inpatient rehabilitation (Table 1).

### 3.2. Neurological Function Recovery

In terms of the neurological function recovery rate, 21.9% of the patients recovered from motor incomplete injury at discharge, whereas 78.1% did not. The recovery rate of patients <50 years of age was 58.0%, and that of those ≥50 years of age was 42.0%. The recovery rates were 79.2% in men and 20.8% in women. Regarding the mechanism of trauma, the recovery rates for vehicle accidents, sports accidents, tumbling, falls from heights, and other causes were 30.3%, 0.4%, 23.4%, 31.6%, and 14.3%, respectively. The neurological function recovery rates of the cervical, thoracic, and lumbosacral spinal cords were 60.2%, 13.4%, and 26.4%, respectively. The recovery rates of neurological function were 66.2% and 33.8% when the times of injury were <24 h and ≥24 h, respectively. Regarding ASIA score at admission, the neurological function recovery rates for ASIA A and ASIA B were 40.3% and 59.7%, respectively. The recovery rate of neurological function without fracture or dislocation was 26.4%, whereas that with fracture or dislocation was 73.6%. The recovery rate of neurological function was 13.0% after conservative treatment and 87.0% after surgical treatment. In terms of surgical procedures, the recovery rate of neurological function was 3.5% for simple spinal cord decompression; 30.3% for decompression and fixation; 63.2% for decompression, fixation, and fusion; and 3.0% for other surgical procedures. The rates of neurological function recovery during hospitalization were 53.7% and 46.3% with and without complications, respectively. The recovery rates of neurological function were 73.6% and 26.4% when the duration of in-hospital stay was <28 d and ≥28 h, respectively, and those of neurological function recovery were 42.9% and 57.1% without and with inpatient rehabilitation, respectively (Table 1).

### 3.3. Univariate and Multivariate Logistic Regression Analysis

There were no significant between-group differences regarding age, sex, injury level, in-hospital complications, surgical procedures, or duration of in-hospital stay (*p* > 0.05). The results of univariate logistic regression analysis revealed that several factors were related to instant neurological recovery in motor complete injury, including mechanism of trauma (*p* = 0.004, odds ratio (OR) = 0.863, 95% confidence interval (CI): 0.779–0.955), ASIA score on admission (A/B) (*p* < 0.001, OR = 5.486, 95% CI: 4.018–7.491), fracture or dislocation (*p* = 0.008, OR = 0.632, 95% CI: 0.449–0.890), treatment protocol (*p* < 0.001, OR = 2.169, 95% CI: 1.432–3.285), and inpatient rehabilitation (*p* < 0.001, OR = 1.930, 95% CI: 1.428–2.611) (Table 2).

**Figure 1:**
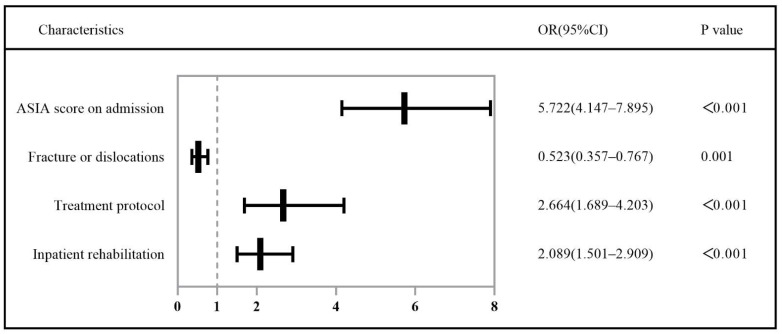
Risk factors for instant recovery of neurological function in patients with motor complete injury in TSCI.

The ASIA score on admission, mechanism of trauma, fracture or dislocation, treatment protocol, and inpatient rehabilitation were reanalyzed using multivariate logistic regression analysis. The results of multiple logistic regression analysis showed that the ASIA score on admission (*p* < 0.001, OR = 5.722, 95% CI: 4.147–7.895), fracture or dislocation (*p* = 0.001, OR = 0.523, 95% CI: 0.357–0.767), treatment protocol (*p* = 0.001, OR = 2.664, 95% CI: 1.689–4.203), and inpatient rehabilitation (*p* < 0.001, OR = 2.089, 95% CI: 1.501–2.909) were independently associated with the recovery of neurological function, as shown in Figure 1.

## 4. Discussion

This study specifically assessed the instant neurological recovery of hospitalized patients with complete motor injury caused by TSCI. We revealed several factors related to instant neurological recovery in patients with motor complete injuries. We found that the ASIA score on admission, fracture or dislocation, treatment protocol, and inpatient rehabilitation were significantly associated with instant neurological recovery in patients. These findings are of value to inform patients and rehabilitation teams to manage expectations of motor and functional recovery.

Previous studies reported that neurological recovery is significantly different among all grades of SCI severity in the following order: C > B > D > A [17]. In the study by Skeers et al. [18], grade-A patients exhibited greater compression than those with motor incomplete injury, and ASIA A was associated with an increased likelihood of severe neurological deficits. In addition, Kirshblum et al. [15] also reported that individuals with incomplete sensory tetraplegia (ASIA B) regain significantly more sensory function than patients with initial ASIA A, which also indirectly indicated that the neurological recovery was worse for patients with initial AISA A than AISA B. Consistent with the findings of previous studies, we found that neurological recovery of patients with initial AISA B injury was significantly better than that of patients with ASIA A. Therefore, we think that timely diagnosis of the severity of neurological impairment is important for functional recovery in TSCI.

Regarding the TSCI with fracture or dislocation, it was reported that the incidence of TSCI is as high as 65.87% [19]. Spinal cord compression caused by TSCI is usually self-evident, mostly due to vertebral fractures or fracture dislocations [20]. TSCI with vertebral fracture or displacement is typically accompanied by substantial spinal cord compression. Previous studies demonstrated that greater cord compression is associated with an increased likelihood of severe neurological deficits following TSCI [18]. In our study, fracture or dislocation was a detrimental factor for neurological improvement. We think that bone fragments from fractures or dislocations could have caused substantial damage to the spinal cord. Shank et al. [7] also suggested that fracture or dislocation injury can result in acute SCI by compromising the spinal canal and causing direct spinal cord compression, which indirectly confirms our results. Therefore, it is particularly important to remove bone and intervertebral disc fragments from the spinal canal and correct the dislocation.

The treatment of TSCI without fractures or dislocations remains controversial. Nakajima recommended conservative treatment for patients with TSCI without fracture or dislocation in a retrospective case-control study [21]. In our study, we found that conservative treatment was an independent risk factor of neurological recovery in patients with ASIA type A/B TSCI compared with surgical treatment. Therefore, we think that patients with TSCI should immediately undergo surgery as treatment, and the relief of spinal cord compression can promote immediate recovery of neurological function to a certain extent. Furthermore, previous studies have revealed that due to spinal cord compression, emergency surgical decompression can break the vicious circle of ischemia–swelling–compression–ischemia, thereby improving the prognosis for recovery of neurological function, which also supports our results [22,23].

Inpatient rehabilitation plays an important role in neurological recovery in patients with TSCI. Research has shown that activity-based interventions, such as transcranial magnetic stimulation, functional electrical stimulation, and robotic-assisted treadmill training, are effective in improving function in individuals with spinal cord injury [24,25]. Nam et al. [26] demonstrated that robot-assisted gait training can restore functional walking and improve locomotor ability, which might enable patients with SCI to increase their level of physical activity and maintain a healthy lifestyle. Our findings suggest that inpatient rehabilitation may be associated with neurological recovery. Xiong et al. [27] also reported that specific acupuncture therapy combined with rehabilitation training could promote neurological recovery in patients with incomplete SCI. Therefore, for patients with TSCI, especially for motor complete injury, rehabilitation therapy plays an irreplaceable role in the recovery of neurological function.

This study has some limitations. We did not analyze all types of ASIA scale conversion in TSCI because we did not consider all TSCI patients, such as those with ASIA C and ASIA D. Although we included a large sample size compared with previous studies, we only focused on the recovery of neurological function of patients in hospitals and conducted analysis while only considering the patient’s condition at the time of discharge. Hence, the applicability of the conclusions drawn is limited to these patients, and care needs to be taken when generalizing these results to other types of patients. In terms of surgical treatment, the patient’s choice to undergo surgery may affect the outcome of neurological recovery, considering that patients had a better recovery of neurological function after the operation, and many other studies have confirmed the benefits of surgery [12,16,28]. Therefore, we strongly recommend that patients with these conditions undergo surgery.

## 5. Conclusions

The prediction of instant neurological recovery based on data available during hospitalization after TSCI is of paramount importance for patients, caregivers, and society. This study confirmed that the ASIA score on admission, fracture or dislocation, treatment protocol, and inpatient rehabilitation were independently associated with the recovery of neurological function, and these predictors have the potential to guide decision making at both the clinical and societal levels.

## Figures and Tables

**Table 1:** Demographic and clinical characteristics of participants.

Characteristics (No., %)	Total Cases (*n* = 1053)	Motor Complete Injury (*n* = 822)	Motor Incomplete Injury (*n* = 231)	*p* Value
Age (years)				0.602
<50	595 (56.5)	461 (56.1)	134 (58.0)	
≥50	458 (43.5)	361 (43.9)	97 (42.0)	
Sex				0.486
Male	851 (80.8)	668 (81.3)	183 (79.2)	
Female	202 (19.2)	154 (18.7)	48 (20.8)	
Mechanism of trauma				0.021
Vehicle accident	257 (24.4)	187 (22.8)	70 (30.3)	
Sport accident	4 (0.5)	3 (0.4)	1 (0.4)	
Tumble	207 (19.6)	153 (18.6)	54 (23.4)	
Fall from height	416 (39.5)	343 (41.7)	73 (31.6)	
Others	169 (16.0)	136 (16.5)	33 (14.3)	
Injury level				<0.001
Cervical spinal cord	571 (54.2)	432 (52.6)	139 (60.2)	
Thoracic spinal cord	254 (24.1)	223 (27.1)	31 (13.4)	
Lumbosacral spinal cord	228 (21.7)	167 (20.3)	61 (26.4)	
Time before admission (h)				0.069
<24	748 (71.0)	595 (72.4)	153 (66.2)	
≥24	305 (29.0)	227 (27.6)	78 (33.8)	
ASIA score on admission				<0.001
A	740 (70.3)	647 (78.7)	93 (40.3)	
B	313 (29.7)	175 (21.3)	138 (59.7)	
Fracture or dislocations				0.008
Without	213 (20.2)	152 (18.5)	61 (26.4)	
With	840 (79.8)	670 (81.5)	170 (73.6)	
Treatment protocol				<0.001
Conservative	231 (21.9)	201 (24.5)	30 (13.0)	
Surgery	822 (78.1)	621 (75.5)	201 (87.0)	
Surgical procedures				0.605
Simple spinal cord decompression	20 (2.4)	13 (2.1)	7 (3.5)	
Decompression and fixation	237 (28.8)	176 (28.3)	61 (30.3)	
Decompression, fixation and fusion	543 (67.1)	416 (70.0)	127 (63.2)	
Other	22 (2.7)	16 (2.6)	6 (3.0)	
In-hospital complications				0.618
Without	550 (52.2)	426 (51.8)	124 (53.7)	
With	503 (47.8)	396 (48.2)	107 (46.3)	
Duration of in-hospital stay (d)				0.264
<28	804 (76.3)	634 (77.1)	170 (73.6)	
≥28	249 (23.7)	188 (22.9)	61 (26.4)	
Inpatient rehabilitation				<0.001
Without	691 (65.6)	592 (72.0)	99 (42.9)	
With	362 (34.4)	230(28.0)	132 (57.1)	

**Table 2:** Univariate logistic regression analysis of variables.

Characteristics	*p*-Value	95% CI	OR
Age	0.602	0.688–1.242	0.924
Sex	0.486	0.791–1.636	1.138
Mechanism of trauma	0.004	0.779–0.955	0.863
Injury level	0.800	0.815–1.171	0.977
In-hospital complications	0.618	0.693–1.244	0.298
Time before admission	0.069	0.978–1.827	1.336
ASIA score on admission (A/B)	<0.001	4.018–7.491	5.486
Fracture or dislocations	0.008	0.449–0.890	0.632
Treatment protocol	<0.001	1.432–3.285	2.169
Surgical procedures	0.338	0.660–1.153	0.872
Duration of in-hospital stay	0.264	0.866–1.691	1.210
Inpatient rehabilitation	<0.001	1.428–2.611	1.930

## Data Availability

The data presented in this study are available on request from the corresponding author. The data are not publicly available due to privacy restrictions.

## Supplementary Materials

The following supporting information can be downloaded at: https://www.mdpi.com/article/10.3390/jcm11144086/s1, Supplementary Material S1: List of ICD-10-CM Codes for Traumatic Spinal Cord Injury (TSCI) Diagnosis; Supplementary Material S2: Data recording form for in-hospital patient with traumatic spinal cord injury in China.
