# Supplementary material for: Factors for Predicting Instant Neurological Recovery of Patients with Motor Complete Traumatic Spinal Cord Injury
Source: J Clin Med. 2022 Jul 14;11(14):4086. doi: 10.3390/jcm11144086 (PMC9319428; doi:10.3390/jcm11144086)
Supplement: Supplementary file 1 [file jcm-11-04086-s001.zip › Supplementary materical/Supplementary material S2.pdf]

Data recording form for in-hospital patient with traumatic spinal cord injury in China

Chinese Association of Orthopaedic Surgeons
中国医师协会 骨科医师分会

Hospital Name____

Hospital Number____

Hospital level: (tertiary A, tertiary B, tertiary C)_____________________

Patient Name____

Hospitalization number____

Patient no.____

Investigator (signature)____

Date of survey____

General Description

1 All case report forms (data recording forms) must be completed with a black ballpoint pen.

2 When you need to choose, please draw a "tick" in the box of the correct option.

3 If necessary, add zeros to the numbers to fill in the spaces, e.g. if the patient number is "14", then put "0014" in the space for the patient number.

4 The format of the date is: day/month/year, for example, April 30, 2001 should be written as 30/04/2001. If necessary, zeroes should also be added to fill in the spaces in front, for example: January 1, 1999 should be written as 01/01/1999. If you do not know the date or part of the date, draw a short horizontal line in that section. If possible, at least the year should be entered.

5 When you need to correct, draw a line on the original number and fill in the correct information nearby. Do not delete the original information with correction fluid or any other method. Fill out the information must be clear. Each change must be signed and dated by the person making the change; do not bracket multiple changes together. If necessary, a brief note should be added to the Notes page to explain why the change is needed. Do not make comments on the edge of the data recording form, comments can only be filled in the Notes page.

6 If some items are not completed as planned during the case excerpting process, please indicate the missing items and the reason for them on the Notes page.

7 The capital of each page of the survey form must be filled in, including the unused pages; however, the unused pages do not need to fill in the date. If the page is useless, draw a straight line on the opposite corner of the page.

8 Please use standard medical abbreviations when completing the data recording form.

9 For each patient entered, the investigator had to sign the first page and the Notes page of the data recording form.

Part I: Basic Information

A1 ID number.

□ □ □ □ □ □ □ □ □ □ □ □ □ □ □ □ □ □

A2 Age:______

A3 Sex: □ 1 male □ 2 female

A4 Place of residence:__________Province__________City__________County

A5 Whether to live in the city where the survey area is located

□ 1 No □ 2 Yes

A6 Occupations

□ 1 officials □ 2 professional and technical personnel □ 3 employees □ 4 enterprise managers □ 5 workers □ 6 farmers □ 7 students □ 8 military personnel □ 9 freelancers □ 10 self-employed persons □ 11 jobless persons □ 12 retired persons □ 13 others

A7 Whether the patient is a first visit or a referral:

□ 1 first consultation □ 2 referral

A8a Phone number 1: □ □ □ □ □ □ □ □ □ □ □

A8b Phone number 2: □ □ □ □ □ □ □ □ □ □ □

Part II: Admission Information

B1a Date of admission to hospital. □ □ day, □ □ month, □ □ □ □ year

B1b Time of admission to the Hospital: □ □ hour

B2 Time from injury to admission to the hospital.

□ □ hour □ □ day

B3a Date of discharge: □ □ day □ □ month □ □ year

B3b Specific time of discharge: □ □ hour

B4 Fee payment method

□ 1 Fully self-funded □ 2 Rural cooperative medical insurance □ 3 Urban residents' medical insurance □ 4 Urban employees' medical insurance □ 5 Commercial insurance □ 6 Poverty relief □ 7 Fully publicly funded □ 8 Other social insurance □ 9 Others.

B5 Discharge methods

口 1 Medical discharge 口 2 Medical transfer 口 3 Non-medical discharge 口 4 Non-medical transfer 口 5 Death 口 6 Other

B6 Whether they received systematic conservative treatment prior to admission to the investigation hospital

口 1 No 口 2 Yes

B7 Whether or not they had undergone spinal surgery prior to admission to the investigating hospital

口 1 No 口 2 Yes

Part III: Injury situation

C1 Etiology of injury.

口 1 Traffic-related etiology (e.g., car accidents, etc.) C2

口 2 Exercise and leisure-related etiologies (e.g., running, swimming, etc.) C2

口 3 Falls, slips and related etiology C2

口 4 Fall from height injuries

口 5 other etiologies C2

C1a Fall height: 口口口. 口 meter

C2 Level of impairment.

口 1 cervical segments 口 2 thoracic segments 口 3 lumbosacral segment

C3 Whether accompanied by spinal fracture or dislocation

口 1 No C5

口 2 Yes

C4 Fracture site:

Serial number	Fracture site		
**C4a**	cervical Spine	口 1 No C4b	口 2 Yes C4a1
**C4b**	thoracic spine	口 1 No C4c	口 2 Yes C4b1
**C4c**	lumbar spine	口 1 No C4d	口 2 Yes C4c1
**C4d**	sacral vertebrae	口 1 No C5	口 2 Yes C5

C4a Fractures occur in which of the following cervical vertebrae specifically and in the

corresponding fracture type.

Serial number	Cervical Segment			Serial number	Type of fracture			
**C4a1**	C1	☐ 1 No...C4a2	☐ 2 Yes	**C4a1.1**	☐ 1 compression fracture	☐ 2 burst fracture	☐ 3 flexion-extension fracture	☐ 4 fracture dislocation
**C4a2**	C2	☐ 1 No...C4a3	☐ 2 Yes	**C4a2.1**	☐ 1 compression fracture	☐ 2 burst fracture	☐ 3 flexion-extension fracture	☐ 4 fracture dislocation
**C4a3**	C3	☐ 1 No...C4a4	☐ 2 Yes	**C4a3.1**	☐ 1 compression fracture	☐ 2 burst fracture	☐ 3 flexion-extension fracture	☐ 4 fracture dislocation
**C4a4**	C4	☐ 1 No...C4a5	☐ 2 Yes	**C4a4.1**	☐ 1 compression fracture	☐ 2 burst fracture	☐ 3 flexion-extension fracture	☐ 4 fracture dislocation
**C4a5**	C4	☐ 1 No...C4a6	☐ 2 Yes	**C4a5.1**	☐ 1 compression fracture	☐ 2 burst fracture	☐ 3 flexion-extension fracture	☐ 4 fracture dislocation
**C4a6**	C6	☐ 1 No...C4a7	☐ 2 Yes	**C4a6.1**	☐ 1 compression fracture	☐ 2 burst fracture	☐ 3 flexion-extension fracture	☐ 4 fracture dislocation
**C4a7**	C7	☐ 1 No...C4b	☐ 2 Yes	**C4a7.1...C4b**	☐ 1 compression fracture	☐ 2 burst fracture	☐ 3 flexion-extension fracture	☐ 4 fracture dislocation

C4b Fractures occur in which of the following thoracic vertebrae specifically and in the corresponding fracture type.

Serial number	Thoracic spine segments			Serial number	Type of fracture			
**C4b1**	T1	☐ 1 No...C4b2	☐ 2 Yes	**C4b1.1**	☐ 1 compression fracture	☐ 2 burst fracture	☐ 3 flexion-extension fracture	☐ 4 fracture dislocation
**C4b2**	T2	☐ 1 No...C4b3	☐ 2 Yes	**C4b2.1**	☐ 1 compression fracture	☐ 2 burst fracture	☐ 3 flexion-extension fracture	☐ 4 fracture dislocation
**C4b3**	T3	☐ 1 No...C4b4	☐ 2 Yes	**C4b3.1**	☐ 1 compression fracture	☐ 2 burst fracture	☐ 3 flexion-extension fracture	☐ 4 fracture dislocation
**C4b4**	T4	☐ 1 No...C4b5	☐ 2 Yes	**C4b4.1**	☐ 1 compression	☐ 2 burst	☐ 3 flexion-extension	☐ 4 fracture

					fracture	fracture	fracture	dislocation
**C4b5**	T5	☐ 1 No...C4b6	☐ 2 Yes	**C4b5.1**	☐ 1 compression fracture	☐ 2 burst fracture	☐ 3 flexion-extension fracture	☐ 4 fracture dislocation
**C4b6**	T6	☐ 1 No...C4b7	☐ 2 Yes	**C4b6.1**	☐ 1 compression fracture	☐ 2 burst fracture	☐ 3 flexion-extension fracture	☐ 4 fracture dislocation
**C4b7**	T7	☐ 1 No...C4b8	☐ 2 Yes	**C4b7.1**	☐ 1 compression fracture	☐ 2 burst fracture	☐ 3 flexion-extension fracture	☐ 4 fracture dislocation
**C4b8**	T8	☐ 1 No...C4b9	☐ 2 Yes	**C4b8.1**	☐ 1 compression fracture	☐ 2 burst fracture	☐ 3 flexion-extension fracture	☐ 4 fracture dislocation
**C4b9**	T9	☐ 1 No...C4b10	☐ 2 Yes	**C4b9.1**	☐ 1 compression fracture	☐ 2 burst fracture	☐ 3 flexion-extension fracture	☐ 4 fracture dislocation
**C4b10**	T10	☐ 1 No...C4b11	☐ 2 Yes	**C4b10.1**	☐ 1 compression fracture	☐ 2 burst fracture	☐ 3 flexion-extension fracture	☐ 4 fracture dislocation
**C4b11**	T11	☐ 1 No...C4b12	☐ 2 Yes	**C4b11.1**	☐ 1 compression fracture	☐ 2 burst fracture	☐ 3 flexion-extension fracture	☐ 4 fracture dislocation
**C4b12**	T12	☐ 1 No...**C4c**	☐ 2 Yes	**C4b12.1...C4c**	☐ 1 compression fracture	☐ 2 burst fracture	☐ 3 flexion-extension fracture	☐ 4 fracture dislocation

C4c Fractures occur in which of the following lumbar vertebrae specifically and in the corresponding fracture type.

Serial number	Lumbar spine segments			Serial number	Type of fracture			
**C4c1**	L1	☐ 1 No...C4c2	☐ 2 Yes	**C4b1.1**	☐ 1 compression fracture	☐ 2 burst fracture	☐ 3 flexion-extension fracture	☐ 4 fracture dislocation
**C4c2**	L2	☐ 1 No...C4c3	☐ 2 Yes	**C4b2.1**	☐ 1 compression fracture	☐ 2 burst fracture	☐ 3 flexion-extension fracture	☐ 4 fracture dislocation
**C4c3**	L3	☐ 1 No...C4c4	☐ 2 Yes	**C4b3.1**	☐ 1 compression fracture	☐ 2 burst fracture	☐ 3 flexion-extension fracture	☐ 4 fracture dislocation
**C4c4**	L4	☐ 1 No C4c5	☐ 2 Yes	**C4b4.1**	☐ 1 compression fracture	☐ 2 burst fracture	☐ 3 flexion-extension fracture	☐ 4 fracture dislocation

**C4c5**	L5	☐ 1 No...**C4d**	☐ 2 Yes	**C4b5.1...C4d**	☐ 1 compression fracture	☐ 2 burst fracture	☐ 3 flexion-extension fracture	☐ 4 fracture dislocation

C5 Degree of damage

☐ 1 complete quadriplegia ☐ 2 incomplete quadriplegia ☐ 3 complete paraplegia ☐ 4 incomplete paraplegia

C6 Presence of compound injuries.

☐ 1 None.........**C7**

☐ 2 Yes

C6a Compound injury situation (check at least one)

☐ 1 Head ☐ 2 Thorax ☐ 3 Abdomen ☐ 4 Extremities ☐ 5 Pelvis

C7 Spinal Cord Injury Score-ASIA on admission to the investigating hospital._

Grading	Function Status
☐ 1	Complete damage with no preserved sensory or motor function in the sacral area
☐ 2	Incomplete damage with sensory function but no motor function below the impairment plane including the sacral segment (S4,5)
☐ 3	Incomplete impairment, with motor function present below the impairment plane and muscle strength of less than grade 3 in more than 50% of the key muscles.
☐ 4	Incomplete impairment, where motor function exists below the impairment plane and where more than 50% of the key muscles have muscle strength greater than or equal to grade 3.
☐ 5	Normal sensory and motor function

Note: If there is no accurate description of clear ASIA classification, it must be assessed by group discussion of the sensory and motor functional status in the case.

Part IV: Surgery, rehabilitation and recovery

D1 Whether to conduct rehabilitation training after spinal cord injury in the investigating hospital

☐ 1 No

☐ 2 Yes

D2 Whether spinal surgery was performed at the investigating hospital

□ 1 No **D5**

□ 2 Yes

D3 Surgical method

□ 1 Simple spinal cord decompression □ 2 Decompression and fixation □ 3. Decompression and fixation fusion □ 4 Other surgical procedures

D4 Surgical approach

□ 1 anterior □ 2 posterior □ 3 combined anterior and posterior □ 4 others

D5 Date of surgery: □□ day □□ month □□□□ year

Specific time of surgery: □□ hour

D6 ASIA score for spinal cord injury at discharge

Grading	Function Status
□ 1	Complete damage with no preserved sensory or motor function in the sacral area
□ 2	Incomplete damage with sensory function but no motor function below the impairment plane including the sacral segment (S4,5)
□ 3	Incomplete impairment, with motor function present below the impairment plane and muscle strength of less than grade 3 in more than 50% of the key muscles.
□ 4	Incomplete impairment, where motor function exists below the impairment plane and where more than 50% of the key muscles have muscle strength greater than or equal to grade 3.
□ 5	Normal sensory and motor function

D7 Presence of complications during hospitalization

□ 1 no complications.....................**D8**

□ 2 with complications

D7a Complications during hospitalization (check at least one)

□ 1 urinary system □ 2 respiratory system □ 3 digestive system □ 4 electrolyte disorders □ 5 decubitus ulcers □ 6 lower extremity venous thrombosis □ 7 cardiovascular system □ 8 other

D8 Presence of nosocomial infection

□ 1 No **D9**

☐ 2 Yes

D8a Site of nosocomial infection (check at least one)

☐ 1 upper respiratory tract ☐ 2 lower respiratory tract ☐ 3 urinary tract ☐ 4 skin and soft tissues ☐ 5 bones and joints ☐ 6 Superficial incisional infection ☐ 7 Deep incisional tissue infection ☐ 8 Organ and cavity infection ☐ 9 Other

D9 Whether death occurs during hospitalization

☐ 1 No **.. End of data recording form**

☐ 2 Yes

D10 Causes of death during hospitalization

☐ 1 respiratory dysfunction ☐ 2 infection ☐ 3 cardiovascular disease ☐ 4 other (please fill in)____

Notes page

Investigator (signature): Date:

Auditor (signature): Date:
